# The humanistic burden of Pompe disease: are there still unmet needs? A systematic review

**DOI:** 10.1186/s12883-017-0983-2

**Published:** 2017-11-22

**Authors:** Benedikt Schoser, Deborah A. Bilder, David Dimmock, Digant Gupta, Emma S. James, Suyash Prasad

**Affiliations:** 1 Friedrich-Baur-Institut, Neurologische Klinik und Poliklinik, Klinikum der Universität München, Ludwig-Maximilians-Universität München, Ziemssenstr, D-80336 Munich, Germany; 2 Department of Psychiatry, Division of Child and Adolescent Psychiatry, University of Utah School of Medicine, Salt Lake City, UT USA; 3 Rady Children’s Institute for Genomic Medicine, San Diego, CA 92123 USA; 4 Bridge Medical Consulting Ltd, Gainsborough House, 2 Sheen Road, Richmond, London, TW9 1AE UK; 5 Audentes Therapeutics, 600 California Street, Floor 17, San Francisco, CA 94104 USA

**Keywords:** Pompe, Humanistic burden, Quality of life, Daily living, Caregiver burden

## Abstract

**Background:**

Humanistic burden considers the impact of an illness on a patient’s health-related quality of life (HRQoL), activities of daily living (ADL), caregiver health, and caregiver QoL. Humanistic burden also considers treatment satisfaction and adherence to treatment regimens. Pompe disease is an autosomal recessive, progressive, multisystemic neuromuscular disease. Approval of enzyme-replacement therapy (ERT) markedly improved prognosis for patients, but considerable morbidity and a substantial humanistic burden remain. This article characterizes the humanistic burden of Pompe disease through a systematic literature review.

**Methods:**

A systematic search of MEDLINE® and Embase® with back-referencing and supplementary literature searches was performed to retrieve data from interventional and non-interventional studies on the humanistic burden of Pompe disease. Publications were screened according to predefined criteria, extracted, and assessed for quality. Extracted data were narratively synthesized.

**Results:**

No publications on the humanistic burden of infantile-onset Pompe disease (IOPD) were identified. As such, of 17 publications included here, all are in patients with late-onset Pompe disease (LOPD). Thirteen publications were initiated after approval of ERT, two were initiated before, and two overlapped the approval of ERT. The review shows that LOPD patients have a significantly lower HRQoL than the general population, even if treated with ERT. On transitioning to ERT, treatment was associated with improvement in the physical component score of the SF-36 and fatigue, although the SF-36 mental component score remained stable. Physical HRQoL remained below population norms after 4 years of ERT. Significantly more ERT-treated patients reported pain than controls, and bodily pain worsened in later years following ERT initiation. Treatment-naïve LOPD patients had significantly poorer ADL functioning compared with the general population, although ERT stabilized deteriorating functioning impairment. ERT studies showed caregivers provide 17.7 h/week informal care on average. Fifty percent, 40% and <20% of caregivers reported mental health, physical health, and financial/relational problems, respectively. In ERT-naïve patients, wheelchair use and home ventilatory support was associated with lower physical HRQoL and ADL functioning. In ERT-treated patients, key factors predicting worse HRQoL and ADL functioning were higher respiratory distress, poorer sleep quality, greater pain, and more fatigue.

**Conclusions:**

Pompe disease has a substantial humanistic burden, with strong inter-relationships among and between humanistic burden parameters and clinical progression.

## Background

The humanistic burden considers the impact of an illness on a patient’s health-related quality of life (HRQoL), activities of daily living (ADL), caregiver health, and caregiver QoL. The humanistic burden also takes into account patients’ treatment satisfaction and adherence to their specific treatment regimen.

Pompe disease is an autosomal recessive, progressive, debilitating disease in which acid α-glucosidase (GAA) deficiency leads to intralysosomal accumulations of glycogen in all tissues. All muscle groups are affected, including ventilatory, cardiac, and skeletal muscles, and there is some evidence of motor neuron dysfunction and pathology of the central and peripheral nervous systems [1–3], with resultant effects on cognitive function, hearing loss, speech muscle pathology, and fine motor dysfunction in patients with classic-infantile Pompe disease.

Pompe disease presents as a wide spectrum of phenotypes, ranging from the severe, rapidly progressive infantile-onset Pompe disease (IOPD) to the more slowly progressing late-onset Pompe disease (LOPD) that can manifest any time from early childhood to late adulthood [4–6].

Clinically, infants with IOPD typically present during the first few weeks of life with hypotonia, progressive weakness, macroglossia, hepatomegaly, and hypertrophic cardiomyopathy. This presentation usually facilitates identification of the disorder [7, 8]; however, identifying LOPD can be more challenging as these patients generally present with slowly progressive limb girdle-type weakness and ventilatory insufficiency without significant cardiomyopathy. Cardiac involvement in LOPD has been described, and may manifest as Wolff-Parkinson-White syndrome, left ventricular hypertrophy, and dilatation of the ascending aorta [9, 10]. Rigid spine syndrome, scoliosis, and low body weight have also been reported in some patients with LOPD that have onset in adolescence, resulting in postural anomalies [7–9]. The laboratory diagnosis of Pompe disease depends on the measurement of GAA activity in the blood or tissues, or the detection of disease-causing variants in the *GAA* gene.

Pompe disease is a rare condition. The reported prevalence estimates vary widely depending on ethnicity and diagnostic approach used [5, 11], and there is a lack of accurate, consistent estimates for both IOPD and LOPD in the published literature; however, for Europe the estimated prevalence of Pompe disease is about 1 in 283,000 [11]. Based on predictions from carrier frequencies, newborn screening data, and retrospectively registered data, the frequency of IOPD has been estimated between 1 in 8684 to 138,000 worldwide [12–14].

Currently, the only specific drug treatment for Pompe disease is ERT with recombinant human GAA (rhGAA), which was approved for use in the United States (US) and Europe in 2006 [15]. A recent systematic review and meta-analysis demonstrated a nearly five-fold reduction in mortality rates over an average study duration of approximately 46 months in patients treated with ERT compared with untreated patients (rate ratio: 0.21; 95% credible interval [CrI]: 0.11–0.41) [6]. This meta-analysis also reported a benefit of ERT in terms of slowing pulmonary functioning decline and improving 6-min walk test (6MWT; the distance an individual is able to walk in 6 min) results. While ERT as the principal treatment targeting the underlying pathology in Pompe disease has transformed the lives of patients, supportive care has an important ongoing role in the management of the condition.

Several limitations of ERT remain, including (1) dependency on high infusion volume, time-consuming infusions at least every other week [4, 15], (2) associated adverse infusion reactions in some patients [4, 15], (3) the inability to clear neuronal glycogen, with subsequent pathology progression and functional decline [16–18]; (4) the reappearance of glycogen in satellite cells [16–18]; (5) continued autophagic build-up in tissue [19]; and (6) antibody responses to ERT that mitigate the efficacy of ERT [20]. These limitations, along with the non-recoverable muscle function loss from Pompe disease, lead to a substantial burden of illness, both for treated and untreated patients.

When investigating the overall burden of a disease, clinical manifestations and economic costs tend to capture the greatest interest of clinicians and payers and garner the focus of most research effort. However, the humanistic burden of a disease, rather than its clinical and economic burden, is typically of greatest concern to patients and their caregivers. Although several reviews have previously been published on the clinical burden of Pompe disease [4–6, 8], the overall humanistic burden has been neglected. This article aims to characterize all aspects of the humanistic burden of Pompe disease and of ERT through a systematic review of the relevant literature for both IOPD and LOPD.

## Methods

No ethical approval was required because no individual patient identifiers were disclosed in the publications contained in this systematic literature review. A PRISMA 2009 Checklist [21] and a review protocol summary are provided as Additional Information.

The following research questions were formulated by the authors at the start of this analysis:

- What is the impact of Pompe disease on humanistic burden in patients receiving ERT throughout the course of clinical studies (both observational and interventional), and in patients transitioning from supportive treatment to ERT during the course of a clinical study?
- What inter-relationships exist among humanistic burden parameters in Pompe disease?
- How does the humanistic burden change as the disease progresses?
- What is the impact of ERT on the humanistic burden of Pompe disease?

### Search strategy

Systematic searches were performed in Embase®/MEDLINE® using Embase.com® to identify relevant articles published through July 2016 on the humanistic burden (patient HRQoL, ADL, caregiver burden, treatment satisfaction and adherence) of Pompe disease. Each search was conducted using controlled vocabulary and key words, and was limited to articles published in English and involving human subjects (as is standard practice for literature reviews of this kind). The specific search terms used are provided as Additional Information. Additional publications were identified through bibliography reviews of relevant articles as well as supplementary searches using Google, Google Scholar and other web-based sources.

### Selection criteria

Titles and abstracts of articles identified were carefully screened by the authors in the initial review for relevance to the topic. Articles were selected for inclusion based on predefined acceptance criteria, which included patient population (adults/children diagnosed with Pompe disease), outcome measures of interest (HRQoL, ADL, caregiver burden, treatment satisfaction, and treatment adherence), and study design (quantitative and/or qualitative data collection). Articles exclusively reporting clinical and/or economic outcomes were excluded, along with those in a non-English language and with an absence of peer-review for articles, editorials, correspondence, and conference abstracts.

Potentially relevant articles were obtained in full-text for further evaluation. Each was screened and its eligibility confirmed by two reviewers. Inconsistencies were resolved through consensus.

### Quality assessment

A descriptive analysis of each publication was conducted during data extraction. Each publication was assessed for quality by considering characteristics that could introduce bias, using the following tools: 1) Newcastle-Ottawa Scale for cohort studies and case-control studies [22]; 2) Adapted Newcastle-Ottawa Scale for cross-sectional studies [22]; 3) AMSTAR measurement tool for reviews [23]; 4) Cochrane risk of bias assessment tool for randomized controlled trials [24].

### Data extraction

Data from the included studies were extracted into a predefined extraction grid by a single reviewer. Information was recorded for study design, setting, patient characteristics, outcome measures, key results, and conclusions. A description of the outcome scales used in the studies included in this review is provided in Table 1. Given the descriptive nature of this systematic review, the extracted data were narratively synthesized.

**Table 1:** Description of outcome scales used in studies in this review

Scale	Description	Range	Interpretation
Brief pain inventory (BPI)	Measures both the intensity of pain (sensory dimension) and interference of pain in the patient’s life (reactive dimension); it also queries the patient about pain relief, pain quality, and patient perception of the cause of pain	Scores range from 0 (no pain) to 10 (pain as bad as you can imagine)	A higher score indicates greater pain
Pain interference score (PIS)	Average score of four items on the BPI devoted to severity of pain	Scores range from 0 (does not interfere) to 10 (completely interferes)	A higher score indicates greater interference of pain
Pain severity score (PSS)	Calculated on the basis of the average interference of pain (assessed on the BPI) with the following seven activities: general activities, mood, walking ability, normal work, relations with other people, sleep, and enjoyment of life	Scores range from 0 (no pain) to 10 (pain as bad as you can imagine)	A higher score indicates greater pain
CarerQoL	Measure of care-related QoL in informal caregivers; comprising two parts: a description of the care situation on seven burden dimensions (CarerQoL-7D) and a valuation component in terms of general QoL using a Visual Analog Scale (CarerQol-VAS)	Assesses the overall well-being of the caregiver on a 0–10 scale, ranging from “completely unhappy” (0) to “completely happy” (10)	A higher score indicates greater happiness
Fatigue severity scale (FSS)	Self-reported nine-item questionnaire concerning the respondent’s fatigue; for example, how fatigue affects motivation, exercise, physical functioning, carrying out duties, interfering with work, family or social life	Participants grade each question on a Likert scale from 1 to 7, where 1 indicates strong disagreement and 7 indicates strong agreement	A higher score indicates more severe fatigue. Scores above 4 indicate significant fatigue and scores above 5 indicate severe fatigue
Hospital anxiety and depression scale (HADS)	14-item self-rated scale. Seven of the items relate to anxiety and seven relate to depression. Each item is rated from 0 to 3, where 0 = not at all to 3 = most severe	Depression and anxiety scores can each range from 0 to 21	Higher scores indicate greater anxiety or depression
Nottingham health profile (NHP)	Assesses subjective health status	Ranges from 0 (good) to 100 (poor)	Higher scores indicate poorer health status
Pittsburgh sleep quality index (PSQI)	19 self-rated questions and five questions rated by the partner. The latter five questions are used for clinical information only and are not tabulated in the scoring of the PSQI. The 19 self-rated questions assess a wide variety of factors relating to sleep quality, including estimates of sleep duration and latency, and the frequency and severity of specific sleep-related problems	Global PSQI score is calculated from the responses given using a predefined algorithm, and ranges from 0 to 21	A higher score indicates worse sleep quality
Rotterdam handicap scale (RHS)	Used to measure a patient’s functional ability and level of handicap, and can be used to monitor a patient’s status over time as well as to evaluate the effectiveness of interventions	Scores per item range from 1 (‘unable to fulfill the task or activity’) to 4 (‘complete fulfillment of the task or activity’). The total score is derived by adding individual component scores and ranges from 9 (‘unable to fulfill any task/activity’) to 36 (‘able to fulfill all applicable tasks or activities’)	A higher score indicates better functional ability
Short-form-36 (SF-36)	Multidimensional HRQoL instrument comprising four physical health scales (physical functioning, role limitations due to physical problems, bodily pain, and general health perceptions) and four mental health scales (vitality, social functioning, role limitations due to emotional problems, and mental health). These eight scales can be aggregated into two summary measures: the physical (PCS) and mental (MCS) component summary scores	Each scale is directly transformed into a 0–100 scale on the assumption that each question carries equal weight	A lower score indicates greater disability (i.e., a score of zero is equivalent to maximum disability and a score of 100 is equivalent to no disability)

## Results

### Overview

**Fig. 1:**
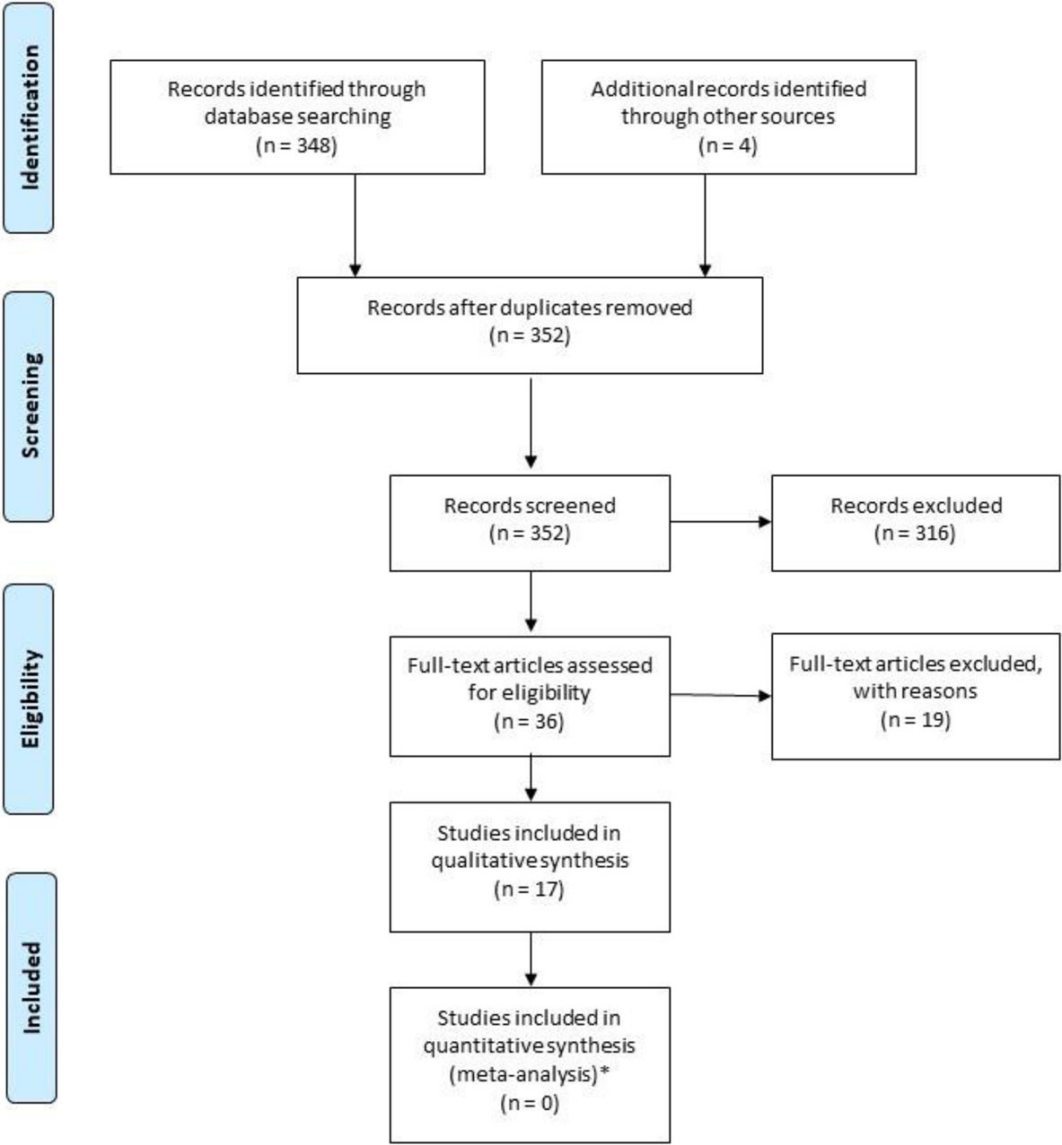
Flowchart of screening and identification process

Titles and abstracts of a total of 352 citations were screened following the initial searches, 36 of which were included for full-text screening. Seventeen articles were subsequently selected for final inclusion in the narrative synthesis (Fig. 1). The basic characteristics and quality ratings of the included studies reporting data on the humanistic burden of Pompe disease are shown in Table 2. All 17 publications included patients with LOPD. Importantly, no studies on the humanistic burden of IOPD were identified, demonstrating a gap in the literature. The 17 LOPD publications included six prospective cohort studies [25–30], one randomized controlled trial (RCT) [31], eight cross-sectional studies [32–39], and two case series [40, 41].

**Table 2:** Basic characteristics of studies reporting on humanistic burden of LOPD

Publication	Quality rating	Study design	Geographic region	Population (sample size)	Disease severity	ERT status
Angelini 2009 [25]	5/9	Prospective cohort	Italy	Symptomatic LOPD patients recruited from three collaborating University Centers (*n* = 11)	Symptomatic (≥2 on the Gardner-Medwin and Walton functional scale)	ERT
Aslan 2016 [26]	6/9	Prospective cohort	Turkey	Subjects with a genetically confirmed diagnosis of LOPD (*n* = 8)	Not stated	ERT
Boentert 2015 [34]	7/9	Cross-sectional	Germany	LOPD patients recruited from specialized outpatient clinics at three neuromuscular centers (*n* = 65)	Not stated (outpatients)	ERT
Freedman 2013 [35]	3/9	Cross-sectional	Australia	LSD patients selected through purposive criterion sampling representing patients receiving ERT to treat a LSD, siblings and parents (9 parents, 4 patients, 3 siblings)	Not stated	ERT
Furusawa 2012 [40]	NA	Case series	Japan	Patients with LOPD who had undergone ERT at the National Center Hospital (*n* = 5)	Wheelchair bound (n = 4) or able to stand for a few minutes (n = 1)	ERT
Güngör 2013 [36]	8/9	Cross-sectional	Germany, Netherlands	LOPD patients recruited through the German patient organization or through Erasmus MC in The Netherlands (*n* = 124)	Full range of severities (mild to fully wheelchair/ventilator dependent)	ERT
Güngör 2016 [29]	3/9	Prospective cohort	Australia, Canada, France, Germany, Netherlands, US, UK, others	Child and adult Pompe disease patients included through national support groups (*n* = 174)	Full range of severities (mild to fully wheelchair/ventilator dependent)	ERT-transition (minimum 6 months follow-up before and after ERT)
Hagemans 2007 [33]	5/9	Cross-sectional	Multiple countries	LOPD patients recruited through patient organizations affiliated with the IPA (*n* = 257)	Full range of severities (mild to fully wheelchair/ventilator dependent)	No ERT
Hagemans 2004 [32]	5/9	Cross-sectional	Australia, Germany, the Netherlands, the UK, the US	LOPD (*n* = 210)	Full range of severities (mild to fully wheelchair/ventilator dependent)	No ERT
Kanters 2013 [37]	5/9	Cross-sectional	Netherlands	All patients at the Center for Lysosomal and Metabolic diseases in Rotterdam receiving informal care plus one caregiver per patient (67 patients; 67 informal caregivers)	Range	ERT
Karabul 2014a [38]	3/9	Cross-sectional	UK	Adult LOPD patients (*n* = 25)	Not stated	ERT
Karabul 2014b [39]	6/9	Cross-sectional	Germany	Adult LOPD patients (*n* = 73)	Not stated	ERT
Regnery 2012 [27]	5/9	Prospective cohort	Germany	Adult LOPD patients from university-based centers (*n* = 38)	Various	ERT
Strothotte 2010 [28]	6/9	Prospective cohort	Germany	LOPD patients treated in participating German university-based centers (*n* = 44)	Various	ERT
Van Capelle 2008 [41]	5/9	Case series	Netherlands	Two severely affected LOPD patients and one moderately affected LOPD patient (*n* = 3)	Moderate (*n* = 1) to severe (*n* = 2)	ERT
van der Meijden 2015 [30]	7/9	Prospective cohort	Australia, Canada, France, Germany, Netherlands, US, UK, others	Child and adult Pompe disease patients included through national support groups (*n* = 408)	Full range of severities (mild to fully wheelchair/ventilator dependent)	ERT-transition
van der Ploeg 2010 [31]	7/9	RCT	US and Europe (eight centers)	LOPD patients, 8 years of age or older, ambulatory, and free of invasive ventilation (*n* = 90)	Range (excluded if invasive ventilation or if required noninvasive ventilation while awake and upright)	ERT

‘ERT-transition’ studies refer to those initiated before the approval of ERT but continuing into the post-ERT era

Quality rating refers to a quality score assigned to each publication considering characteristics that could introduce bias, using the following tools: 1) Newcastle-Ottawa Scale for cohort studies and case-control studies [22]; 2) Adapted Newcastle-Ottawa Scale for cross-sectional studies [22]; 3) AMSTAR measurement tool for reviews [23]; 4) Cochrane risk of bias assessment tool for randomized controlled trials [24]. The maximum score for the Newcastle-Ottawa Scale is 9

IPA: International Pompe Association; LOPD: Late-onset Pompe disease; LSD: Lysosomal storage disorders; MC: Medical center; RCT: Randomized controlled trial

Five of the 17 publications are based on separate analyses of the International Pompe Survey, a long-term prospective natural history study initiated 4 years prior to the approval of ERT and continuing today [29, 30, 32, 33, 36]. This survey was established to collect information on the course of the disease and its burden on patients, and includes patients from Australia, Germany, the Netherlands, the United Kingdom (UK), and the United States of America (US).

Thirteen publications included in this systematic review are based on clinical studies that were initiated after the approval of ERT [25–28, 31, 34–41]; consequently, all participants were receiving open-labeled ERT throughout the course of these studies. Two publications published before ERT approval describe participants treated in the absence of ERT [32, 33]. Finally, two publications [29, 30] are based on studies that were initiated before ERT approval and continued into the ERT era; therefore, these studies include patients who were not receiving ERT at study onset but transitioned to ERT during the study.

In addition to the complete absence of publications reporting on the humanistic burden of IOPD, this review did not identify any LOPD studies describing treatment satisfaction or adherence. Therefore, the following analysis focuses on the impact of LOPD on humanistic burden, and is structured according to the predefined research questions outlined above.

### LOPD is associated with substantial humanistic burden

This section focuses on the overall reported humanistic burden of LOPD, both in the absence and presence of ERT.

#### HRQoL

Overall HRQoL burden was described in four studies; one in the absence of ERT [32], two among patients treated with ERT [26, 34], and one RCT in which patients were randomized to either placebo or ERT [31]. HRQoL was typically measured using the 36-item Short-Form Survey (SF-36) (Table 1).

Hagemans et al. [32] reported a cross-sectional analysis of the International Pompe Survey in 210 patients with LOPD, prior to approval of ERT. Patients presented with a wide range of disease severity, from the mildly affected to wheelchair- and ventilator-dependent, and reported significantly poorer HRQoL on the physical functioning, physical role functioning, general health, vitality, and social functioning subscales of the SF-36 than the general population (*p* < 0.001 on all scales). The difference was most profound for the physical functioning scale, with an adjusted mean score among patients of 29.3, compared with 83.1 (standard deviation [SD] not reported) in the general population. No significant differences were found for the bodily pain, emotional role functioning, or mental health scales, which, the authors speculated may have reflected the slow progression of limitations in daily activities, allowing patients to adapt over time to their situation and adjust their expectations. In a subset of 38 Dutch patients, the authors compared SF-36 scores at baseline and after 1 year; participants were asked on a 5-point scale whether their physical situation had improved a lot, improved a little, remained the same, deteriorated a little, or deteriorated a lot. Twenty-seven of the 38 participants reported deterioration in their physical situation, while none reported improvement. No significant differences were seen for any of the SF-36 subscales between the two time points. The authors suggested that either changes in the physical situation were not always accompanied by changes in QoL or that, as a generic QoL instrument, the SF-36 was insufficiently sensitive to capture such changes.

van der Ploeg et al. [31] reported data from the Late Onset Treatment Study (LOTS), an RCT of 90 LOPD patients aged 8 years or older, ambulatory, and free of invasive ventilation, who received ERT or placebo for 78 weeks. All patients had substantially diminished SF-36 physical component summary (PCS) scores at baseline (ERT-treated patients, 34.3 ± 8.9; placebo-treated patients, 34.9 ± 7.2), which were more than 1.5 SD below the norm for the US general population (50 ± 10). Importantly, despite treatment with ERT, the mean SF-36 PCS score did not increase significantly over the 78-week study ([35.1 ± 9.9], change [95% CI], 0.80 [−1.22 to 2.82]).

Boentert et al. [34] presented a cross-sectional analysis in which 60 of 65 German patients received ERT (mean ERT duration, 5 years), while Aslan et al. [26] described a prospective cohort study of nine Turkish LOPD patients receiving ERT, who participated in an inspiratory muscle training (IMT) program to improve QoL. Boentert et al. stated (without providing quantitative data) that despite ERT, LOPD patients had significantly lower HRQoL than the general population, based on the SF-36 subscales for physical functioning, physical role limitation, general health perception, vitality and social functioning. Aslan et al. also reported impaired HRQoL despite ERT, using the Nottingham Health Profile (NHP), which assesses subjective health status on a scale from 0 (good) to 100 (poor). At baseline, subscales of the NHP ranged from 15.7 for emotional reaction to 82.8 for physical ability. Following implementation of IMT, this study reported improvement in social isolation from 22.5 (interquartile range, 53.8–100.0) to 0.0 (0.0–16.9) (*p* = 0.02), but no other HRQoL subscale demonstrated a treatment response. This led the authors to speculate that participation in the IMT program and the associated weekly telephone contact may have been responsible for the improved social isolation scores.

#### Patient-reported symptoms

This section describes patient-reported symptoms (PRS) that were reported alongside HRQoL, ADL, caregiver burden, treatment satisfaction and adherence. This is included because studies reporting on humanistic parameters frequently also describe common symptoms of Pompe disease (most notably pain, fatigue, and sleep disorders) with a considerable negative impact on humanistic burden. A systematic review of PRS was beyond the protocol-defined objectives of this review because PRS is typically classified under clinical burden.

Patient-reported symptoms were discussed in two publications in the absence of ERT [30, 32], and five publications in the presence of ERT [26, 34, 36, 38, 39]. In Hagemans et al. [32], where no ERT was available, excessive daytime sleepiness (EDS) was reported in approximately 25% of patients and sleep disturbances were reported in more than 40% of patients. In a later analysis of the same study, van der Meijden et al. [30] found that 78% of patients experienced fatigue (Fatigue Severity Scale [FSS] score ≥ 4), and 67% reported severe fatigue (FSS score ≥ 5).

Patients receiving ERT also had poor sleep quality, as assessed by Aslan et al. [26] using the Pittsburgh Sleep Quality Index (PSQI) to measure the quality and patterns of sleep during the previous four weeks. Among these patients, there was a median (interquartile range) total score of 5.0 (2.0–10.5; a cut off score of 5 or more distinguishes ‘bad sleepers’). Introduction of IMT did not improve sleep quality. Boentert et al. [34] also reported sleep symptoms, noting that 45.2% of patients were bad sleepers according to the PSQI (mean PSQI global score, 6.2 ± 3.7). Scores for sleep disturbances, sleep quality impairment and reduced daytime performance were the highest of the PSQI component scales. EDS was reported by 24.6% of patients (mean ESS, 6.9 ± 4.3).

In addition, Boentert et al. also presented respiratory symptoms, reporting that morning headache was reported by 40.0% of patients (‘occasional’ 35.4%; ‘often’ 4.6%) [34]. Nocturnal dyspnea was specified as ‘occasional’ by 18.5% of patients and as ‘often’ by 6.2%. Orthopnea was reported by 60.0% of patients, resting dyspnea in the upright position by 4.6%, and exertional dyspnea by 93.8% of patients. Dyspnea was reported in 29.2%, 40.0% and 24.6% of patients on mild, moderate and heavy exercise, respectively.

A cross-sectional study in 57 German adult LOPD patients (84% on ERT) and 57 controls reported the impact of urge incontinence and gastrointestinal symptoms [39]. In this study, stool urgency, diarrhea, and urinary urge incontinence were reported significantly more frequently in patients compared with the age- and gender-matched controls (55%, 56%, 33% vs. 20%, 18%, 7%).

In a cross-sectional analysis from Germany and the Netherlands from the International Pompe Survey, Güngör et al. [36] assessed the presence and severity of pain in 124 adult LOPD patients, 101 of whom were receiving ERT (median ERT duration, 4 years). They noted that a significantly greater proportion of ERT-treated LOPD patients reported pain compared with unaffected controls (45% vs. 27%, respectively; *p* = 0.004) on the Brief Pain Inventory (BPI) (Short-Form). In this study, the median pain severity score (PSS) – calculated from the BPI (Table 1) – was higher (worse) among patients than controls reporting pain in the previous 24 h (median PSS 3.1 [range 0.75–8] vs. 2.6, [range 0.75–5.25], respectively; *p* = 0.06). Similarly, the pain interference score (PIS; also calculated from BPI), was significantly higher (worse) in patients than controls (median PIS 3.3 [range 0–8.4] vs. 1.3 [range 0–4.4] respectively; *p* = 0.001). Pain particularly interfered (PIS score ≥ 4) with patients’ general activities, walking, and normal work, and showed a mild interference (PIS score ≥ 3) with mood, sleep, and enjoyment of life. For each of the seven domains of daily life, PIS scores were significantly worse in LOPD patients than controls, except for interference with sleep. The authors concluded that while pain is not the most prominent symptom of Pompe disease, it is a common and debilitating aspect of the disease that merits identification and management in this population.

In a follow-up to this study, the same group surveyed 25 patients from the UK using the long-form of BPI, which assesses pain over the previous 7 days [38]. This study reported that 88% of patients reported pain in the last week, a considerably higher proportion than the 45% reporting current pain in Güngör et al. [36]. Median PSS (3.9 [0.5–7.5] vs 3.1 [0.75–8.0]) and median PIS (3.9 [0–7.5] vs 3.3 [0–8.4]) were only slightly higher (worse) in this paper than in the publication by Güngör et al. [36, 38]. These data reinforce the troublesome nature of pain in LOPD.

#### ADL

The International Pompe Survey is the primary source of data on ADL burden [33]. This cross-sectional analysis was conducted in 257 patients with treatment-naïve LOPD (across a broad range of severity) with a median age of onset of 38 years. The impact of LOPD on participation in ADL was assessed using the Rotterdam Handicap Scale (RHS). The mean RHS score among patients in this study was 25.9 ± 6.5 (median, 27), compared with a maximum of 36, which would be scored by a healthy person. Mean RHS scores ranged from 25.5 in Germany to 27.7 in France, but the differences between countries were not statistically significant. Highest scores (reflecting least restrictions) were found for ‘mobility indoors’ and ‘leisure activities indoors’, while the lowest scores were found for ‘domestic tasks indoors’, ‘domestic tasks outdoors’, and ‘work/study’. The authors suggested that their findings demonstrate the considerable impact of Pompe disease on patients’ everyday life experiences.

#### Caregiver burden

One publication so far examined caregiver burden in LOPD [37]. This Dutch study was conducted among 67 patients with LOPD and their caregivers. Caregivers were most often partners (92%), while for children, parents were generally the primary caregivers (94%). Fifty-nine of the 67 patients were receiving ERT.

Caregivers provided about 17.7 h per week of informal care, with some variation (SD 27.5) [37]. Adults primarily received maintenance for household activities (48% of time) and only limited for personal care (18%). In pediatric patients, informal care given as personal care (32% of time) and social activities (36%) was most significant. No caregiver time was spent on specific nursing activities in both age groups. Informal care was provided at the expense of the caregiver’s leisure or work time. The majority (97%) of caregivers gained ‘some satisfaction’, while up to 60% gained a ‘lot of fulfillment’ for providing care. 50% of caregivers reported mental health problems, while 40% had physical health problems. The average burden for caregivers was 3.2 (range, 0.0 to 8.9), measured on a self-rated Visual Analog Scale (VAS, ranging from 0 [not at all straining] to 10 [too much straining]). The mean burden of caregiving for patients below the age of 18 years was higher than for adults (4.1 versus 2.9 respectively; *p* = 0.075). Caregiver burden for patients with LOPD was relatively mild and well-being was not far below the population norms.

### Strong inter-relationships exist among humanistic burden parameters

Beyond humanistic burden overall, the association between individual parameters of humanistic burden was also investigated. Data on these relationships in the absence and presence of ERT are taken primarily from the four studies, previously described [33, 34, 36, 37].

#### Poorer HRQoL is associated with increased fatigue, reduced sleep quality, and increased pain

In Boentert et al. [34], fatigue among ERT-treated LOPD patients was assessed using the FSS and sleep quality was measured using the PSQI. In this analysis, the PCS and MCS of the SF-36 were both inversely correlated with the FSS score (PCS, *r* = −0.46, *p* < 0.001; MCS, *r* = −0.44, *p* < 0.01) and the PSQI global score (PCS, *r* = −0.38, *p* < 0.001; MCS, −0.47, p < 0.001). In terms of the impact of pain on HRQoL, Güngör et al. [36] reported that ERT-treated patients with pain had significantly lower physical (median PCS score 30 [range, 11–45] vs. 35 [range, 17–58], respectively; p < 0.001) and mental HRQoL (median MCS scores 54 [range, 29–74] vs. 58 [range, 29–71], respectively; *p* = 0.049) than those without pain.

#### Poorer HRQoL (except the mental health domain) is associated with greater functional disability

The relationship between the RHS and the SF-36 was investigated in the absence of ERT by Hagemans et al. [33]. The RHS correlated significantly with all SF-36 subscales except for the mental health domain. The largest positive correlation of RHS (*r* = 0.83, *p* < 0.001) was found with the physical functioning subscale of SF-36.

#### Poorer HRQoL is associated with greater caregiver burden

Kanters et al. [37] reported a significant, inverse correlation between patient HRQoL and both caregiver burden and the volume of care provided (rho = −0.430 and rho = −0.465, respectively; *p* < 0.001 in both cases) in ERT-treated patients. Patients with the lowest HRQoL (lowest quartile) received 35.1 h of care per week, with a caregiver burden of 5.3 on the self-rated burden scale, whereas patients with HRQoL in the highest quartile received only 3.9 h of care per week, with a lower caregiver burden of 2.3 on the self-rated scale.

#### Greater functional disability is associated with greater fatigue, poorer sleep quality, and increased pain

Boentert et al. [34] found that the functional ability of ERT-treated patients (measured using RHS) was inversely correlated with fatigue (FSS, *r* = −0.40, *p* < 0.01) and sleep disturbance, (PSQI, *r* = −0.46, p < 0.001). Similarly, Güngör et al. [36] reported that ERT-treated patients with pain had lower functional abilities based on median RHS scores than those without pain (26 [range, 15–36] vs. 28 [range, 16–36], respectively; *p* = 0.02).

#### Greater sleep disturbance is correlated with increased fatigue

Boentert et al. [34] reported, unsurprisingly, that PSQI global score was positively correlated with FSS score (*r* = 0.33, *p* = 0.01) in the presence of ERT, indicating that fatigue increased with greater sleep disturbance.

#### Increased pain is associated with increased depression and anxiety

Güngör et al. [36] found that ERT-treated patients with pain had significantly higher levels of depression and anxiety than those without pain. Anxiety and depression were measured using the median Hospital Anxiety and Depression Scale (HADS), with higher scores indicating greater anxiety or depression. HADS anxiety score among patients reporting pain was 5 (range, 0–15) compared with 3 (range, 0–12) among patients with no pain (*p* = 0.003), while the HADS depression score was 5 (range, 0–13) and 3 (range, 0–12) for patients with and without pain, respectively (*p* = 0.005).

### Clinical progression of disease is strongly associated with greater humanistic burden

We investigated the impact of clinical progression on humanistic burden using data from four studies described previously [32–34, 37].

#### Wheelchair use is associated with decreased HRQoL, greater functional disability, and greater caregiver burden

Hagemans et al. [32] found that wheelchair use is associated with lower HRQoL and ADL scores. In their 2004 study, untreated patients who used a wheelchair scored on average 23.6 points lower on the physical functioning scale and 15.1 points lower on the social functioning scale of the SF-36 than patients who did not need a wheelchair (regression coefficient (B) = −23.6 and −15.1, *p* < 0.001) [32]. In their 2007 study, Hagemans et al. [33] reported significantly lower mean RHS scores in patients who used a wheelchair than those who did not (20.9 vs. 29.5, respectively; p < 0.001).

Kanters et al. [37] reported that ERT-treated wheelchair users received significantly more hours of informal care than patients who were not wheelchair-dependent (25.6 h vs. 14.0 h, respectively; p < 0.001). As such, caregiver burden was significantly higher (self-rated scale, 5.1 vs. 2.4, respectively; *p* = 0.002) and caregiver well-being was significantly lower (CarerQoL-VAS, 6.4 vs. 7.5, respectively; *p* = 0.024) for patients who used a wheelchair than those who did not.

#### Increased respiratory distress is associated with poorer HRQoL, greater functional disability, and greater sleep disturbance and fatigue

An association between increased respiratory symptoms and poorer HRQoL (both physical and mental components) among ERT-treated patients was reported by Boentert et al. [34]. Respiratory symptoms were quantified using the respiratory symptoms questionnaire (RSQ), in which a low score indicates less frequent respiratory symptoms. Both the PCS score and the MCS score were negatively correlated with the RSQ score (PCS, *r* = −0.25, *p* < 0.05; MCS, *r* = −0.46, *p* < 0.001). This finding is consistent with data from untreated patients reported by Hagemans et al. [32], in which reduced respiratory distress was significantly associated with higher HRQoL.

Boentert et al. [34] also found that higher levels of respiratory distress were associated with greater sleep disturbance, as demonstrated by a positive correlation between PSQI and RSQ scores (*r* = 0.43, *p* < 0.0001). Fatigue, measured using the FSS, was also positively associated with respiratory distress, assessed using the RSQ (*r* = 0.58, p < 0.0001), while respiratory distress and functional ability were inversely related, with RHS scores strongly associated with both upright and supine forced vital capacity (FVC) (*r* = 0.62 and *r* = 0.69, respectively, *p* < 0.001). Similar findings were observed in the absence of ERT, with Hagemans et al. [32] reporting that the need for home ventilatory support was associated with lower physical functioning scores (B = −8.4, *p* = 0.004), and that mean RHS scores were significantly lower in patients receiving respiratory support than those without it (22.9 vs. 28.5, p < 0.001). Among the patients who used respiratory support, the correlation between the number of hours of ventilation and the RHS score was −0.59 (p < 0.001), suggesting a reduction in functional ability with increasing use of ventilation. Consistent with this, in this study, almost all patients requiring more than 12 h of ventilation per day required a wheelchair.

#### Cardiopulmonary distress is associated with increased fatigue in male patients

Boentert et al. [34] assessed the relationship between fatigue and “cardiopulmonary distress” in patients receiving ERT, as measured using the 6MWT and FVC. It should be noted that 6MWT and FVC are not direct measures of cardiopulmonary distress; the 6MWT in particular reflects various aspects of physical functioning and may be influenced by cardiac function, walking ability, and respiratory muscle strength. The FSS score was inversely correlated with the 6MWT (*r* = −0.35, *p* < 0.01) (meaning that patients with more severe fatigue performed less well on the 6MWT), but surprisingly, this negative correlation was only apparent in the male subgroup (*r* = −0.53, p < 0.01), indicating that fatigue in female patients may be independent of motor performance.

### ERT is associated with short-term stabilization of humanistic burden

Here, we consider the impact of ERT on the principal aspects of humanistic burden. No data describing the impact of ERT on caregiver burden were identified.

#### HRQoL

Seven articles describe the impact of ERT on HRQoL in LOPD (Table 2) [25, 27–29, 31, 35, 39]: van der Ploeg et al. (2010) reported results from LOTS [31] (discussed above); Güngör et al. [29] evaluated 174 untreated patients who transitioned to ERT during the study; Angelini et al. [25] describe clinical response and changes in functional ability in a prospective cohort study of 11 adult patients; Strothotte et al. [28] and Regnery et al. [27] report data from an open-label observational trial in 44 and 38 adult patients, respectively, in Germany over periods of 12 and 36 months Freedman et al. [35] present a cross-sectional analysis of seven families of young patients (age not specified) with LOPD in Australia, using semi-structured interviews; and van Capelle [41] reports a case series of two children and one adult with LOPD treated with ERT in the Netherlands.

As reported earlier, van der Ploeg et al. found that untreated patients with LOPD had lower SF-36 scores at baseline, which did not improve significantly over 78 weeks’ treatment with ERT [31]. Similarly, Güngör et al. [29], evaluated PCS and MCS components of the SF-36 during three time periods: pre-ERT, first 2 years of ERT, and after more than 2 years of ERT. Before ERT, the PCS decreased significantly by 0.73 score points per year (sp/y). This was followed in the first 2 years after treatment by a significant increase of 1.49 sp./y, and by stabilization thereafter. The MCS was generally stable during the entire follow-up period. Throughout treatment follow-up, the physical functioning, physical role, general health and vitality domains improved, with particularly large increases during the first 2 years of ERT in physical role (9 sp./y), general health (5 sp./y) and vitality (4 sp./y), and a renewed decline thereafter. Mental health domain scores increased in the first 2 years of ERT (2 sp./y), and then declined, while social functioning and emotional role did not improve with treatment. The authors noted that the initiation of ERT halted the progressive decline in physical health status observed in the pre-treatment period, and argued that improvements in the physical functioning and physical role domains may be attributed to the positive effect of ERT on muscle function and strength reported in other studies.

A small prospective Italian study [25], described the clinical response and changes in functional ability in 11 adult patients (age at onset 15–42 years), although results of SF-36 testing are reported in only three patients: one had a mild improvement of mental health but not of bodily pain; the second had no improvement in mental health or role physical; and no HRQoL data are available for the third patient who stopped treatment after 3 months following ERT-related adverse events. These limited data are consistent with the open-label observational study of adult patients (mean age at onset 36 years) in Germany [27, 28]. Here, patients showed no significant longitudinal changes in HRQoL over the course of either 12 or 36 months of ERT treatment [27, 28]. The authors concluded that ERT stabilizes the chronic progressive natural disease course of LOPD in some patients, although motor performance and lung function still appear to decline in up to a third [27, 28].

Freedman et al. [35] reported on a cross-sectional study that used qualitative interviews to assess ERT response. They concluded that improved physical health resulted in increased psychosocial well-being. A benefit of ERT was also found by van Capelle et al. [41] in SF-36 sub-domains for two of three patients for whom these data were reported: one patient showed improvements on the physical health and, to a lesser extent, on the mental health domains, while the other showed particular improvements on the bodily pain and general health domains (no further details provided). The methodology of these studies (e.g., small sample size and reliance on qualitative interviews) limits interpretation and generalizability of their results.

#### Patient-reported symptoms

Data on the impact of ERT on fatigue and pain were identified from the following four publications on humanistic burden, as described above [25, 29, 30, 41].

##### Fatigue

In a 10-year analysis of the International Pompe Survey evaluating 163 patients who transitioned to ERT during the study [30], the proportion of patients reporting fatigue (FSS ≥4) fell from 85% at baseline to 79% at the last follow-up (*p*-value not reported); the proportion who were severely fatigued (FSS ≥5) fell from 68% to 55% (p-value not reported). Fatigue improved mainly in women, patients aged 45 years or older, and those with a disease duration less than 15 years. Improvement in fatigue with length of ERT treatment was also reported by van Capelle et al. [41] among two of three patients studied.

##### Pain

Güngör et al. [29] found that bodily pain worsened in the years prior to ERT initiation, then improved for 2 years during ERT, before declining again after 2 years. Interestingly, the authors also calculated the expected bodily pain scores at three timepoints (4 years before ERT, at start of ERT, and after 4 years of ERT) and reported that the scores were more favorable than population norms at all timepoints. No further details of this analysis are provided. Data from Angelini et al. [25] were available for two of the three patients who completed SF-36 questionnaires; one reported mild improvement in bodily pain on ERT initiation, while the second reported no improvement.

In a clinical survey study, 45% of 124 LOPD patients receiving long-term ERT (median ERT duration, 4 [0.07–12] years) reported having had pain in the previous 24 h, versus 27% of healthy adult controls (*p* = 0.004) [36]. Among patients who reported pain, the median PSS was 3.1 (indicating mild pain) and the median PIS was 3.3 (indicating mild interference); these compare with scores of 2.6 (*p* = 0.06) and 1.3 (*p* = 0.001) for PSS and PIS, respectively, in controls. Relative to patients without pain, those with pain had lower RHS scores (*p* = 0.02), lower SF-36 Physical and Mental component summary scores (*p* < 0.001 and *p* = 0.049), and higher levels of depression and anxiety (*p* = 0.005 and *p* = 0.003). In this largest study on pain, nearly one in two LOPD patients had experienced pain in the previous 24 h. Although pain severity and its interference with daily life was mild, pain was related to a lower QoL, reduced participation in daily life, and greater depression and anxiety. Pain management should therefore be seen as part of clinical practice involving Pompe patients [36].

#### ADL

The impact of ERT on patients’ ability to perform ADL was reported in three studies, including Güngör et al. [29] and van Capelle et al. [41] described previously. In addition, information is taken from a Japanese case series of five long-term, ventilator-dependent patients with advanced LOPD (mean age of onset, 22 years), which examined the efficacy of ERT over a 2-year period [40].

In the International Pompe Survey [29], RHS scores decreased significantly by a mean of 0.49 sp./y (95% CI, −0.63 to −0.34) before ERT initiation and stabilized during ERT treatment (−0.02 sp./y; 95% CI, −0.17 to 0.13). No formal statistical comparisons between the two time periods were provided for ADL. Subgroup analyses showed that overall, RHS scores were better in less severely affected than in more severely affected patients, but the effects of ERT on the ADL of these subgroups did not differ. Van Capelle et al. [41] reported improved RHS scores over time for two of three adults receiving ERT (from 16 and 18 after 3 years of ERT to 25 and 20, respectively, after 8 years). Finally, Furusawa et al. [40] assessed the impact of ERT on patient ADL through interviews with patients and their families. Qualitatively, the authors reported that all patients showed some improvements in ADL and in muscle function, while they also argued that ERT elicited improvements in walking distance and stabilization of pulmonary function. The authors speculated that the improved muscle strength resulted in the better ADL and HRQoL during the follow-up period; however, no quantitative analysis was provided to support this.

## Discussion

### Overview

Pompe disease is a rare and devastating condition for both patients and their families. The clinical burden of Pompe disease, including impact on survival and physical function, has been reviewed elsewhere [4–6, 8]; however, no systematic review on the humanistic burden of Pompe disease has been published. Here, we present the findings from 17 published papers and systematically describe the humanistic burden of Pompe disease.

A major finding of this review is the complete absence of data on the humanistic burden of IOPD, precluding any discussion in this area. Further, although 17 publications describe the humanistic burden of LOPD, only two of these report studies conducted in the absence of ERT, leaving a deficiency in information on untreated patients. However, the association of LOPD with the many measures of clinical, functional, social and emotional well-being that comprise the humanistic burden is reasonably well documented, confirming the impact of LOPD on the everyday life of patients and their caregivers. Although the overall burden of LOPD is substantial for patients, it is interesting to note that the impact on caregivers reported in an isolated study [37] appears to be relatively modest. This may be because families do not consider it burdensome to care for loved ones [42–44], or it may reflect adaptation to their family condition; LOPD is a slowly progressive, heterogeneous disease, and the context may be similar to other chronic disorders or neuromuscular diseases, where caregivers and patients adjust their expectations and priorities over time. This reinforces the need to robustly study the impact of caring on families, and underscores the importance of examining and understanding patients’ and caregivers’ perspectives on the burden of illness, alongside traditional clinical and economic investigations.

**Fig. 2:**
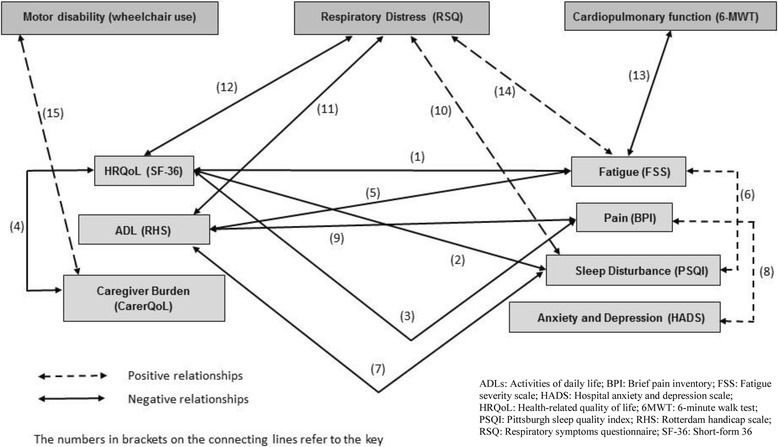
Inter-relationships between clinical, humanistic and economic burden parameters in LOPD patients receiving ERT

Our review demonstrates that clinical disease progression is strongly associated with greater humanistic burden, and also shows strong correlations among individual humanistic burden parameters such as HRQoL, ADL, and caregiver burden. Importantly, ERT does not substantially improve this. Figure 2 (and its accompanying key) provides a graphical illustration of correlations between humanistic and clinical burden parameters in LOPD patients receiving ERT. Unsurprisingly, deterioration in one parameter is strongly linked to deterioration in another, and continues despite ERT. Although ERT provides some benefit, the results appear to be modest, and patients remain below population norms.

This ongoing burden of Pompe disease despite ERT may be partly due, not only to the variety of muscle groups affected, but also to pathology of the CNS and PNS [1–3]. However, presently only brain autopsies in untreated patients have shown clear evidence of glycogen storage in the CNS [45, 46], and periventricular white matter abnormalities on brain magnetic resonance imaging (MRI) have been seen in children receiving ERT [1]. Measuring the effects of CNS pathology on cognitive function is confounded by co-occurring hearing loss, speech muscle pathology, and fine motor dysfunction; however, it is clear from emerging signs and symptoms, which may be caused by neuronal glycogen deposition, that ERT is unable to address all aspects of the disease at least in infants with Pompe disease [3, 47–51]. While ERT clearly slows disease progression for many patients, available data demonstrate that it is limited in its ability to improve and maintain clinical symptomatology and associated disease burden in the long term. The reasons for this are not fully understood, but given that improvements in one facet of the disease burden are expected to positively influence others, there is still substantial potential for enriching patients’ lives beyond the response currently achieved with ERT.

Evidence indicates that ERT leads to short-term improvement and a mid-term stabilization of HRQoL, although results have not been shown to persist in the long-term so far. For example, in one study, there was no improvement in mean SF-36 PCS scores over 78 weeks of treatment with ERT [31], while another found that although ERT reversed a decline in SF-36 PCS, the benefit plateaued after 2 years without normalizing; ERT appears to halt the decline in patients’ ability to perform ADL, but reports on ERT’s association with improved functioning are mixed [29, 40, 41]. As such, gaps remain in providing treatment options that treat the underlying disease, improve clinical symptomatology, and positively affect patients’ abilities to lead normal lives.

It is interesting to note that pain was reported by a number of patients and interfered with their general activities, walking, and normal work, as well as mood, sleep and enjoyment of life [36]. While ERT temporarily stabilized bodily pain, it did not reverse it; in fact, after 2 years of ERT, pain began to worsen once again [29]. The reason for this is not clear, but could be due to neuronal deposition of glycogen, which cannot be cleared by ERT [16, 17]. Given the substantial impact pain has on people’s lives, further investigation is warranted.

### Gaps and limitations

Orphan diseases are hampered by lack of information within the medical community and the scientific literature [52], and this is also true for our topic of review, the humanistic burden of Pompe disease. For the most severely affected patients – those with IOPD – the humanistic burden of is unexplored, preempting any comparisons between IOPD and LOPD. For LOPD, data on the humanistic burden are comparatively extensive, although significant gaps remain. Data were most robust for HRQoL, the majority of which were generated after ERT approval in 2006. There were very limited longitudinal data on the progression of HRQoL, ADL and caregiver burden in LOPD in both the presence and absence of ERT, and no data on change in caregiver burden on the introduction of ERT. In addition, across the entire spectrum of disease severity, there are no data on patient adherence and satisfaction with ERT.

Few studies examining humanistic burden parameters were conducted before marketing approval was granted for ERT, and their limited depth and breadth merit consideration when interpreting this analysis. Similarly, there is no published literature on patients who discontinued ERT therapy, resulting in a lack of understanding of how this affects patients. Data quality also limits this systematic review; due to the rarity of Pompe disease, many of the published studies are small, cross-sectional, and lack the statistical robustness required to draw firm conclusions. In addition, based on the screening criteria employed, publications including relevant data only in the body of the report but not in the title or the abstract may have been overlooked, although attempts were made to mitigate this through back-referencing and supplementary searches. Finally, the possibility of double or triple reported subjects cannot be excluded.

### Avenues for future research

The introduction of ERT into the Pompe disease treatment paradigm has reduced mortality and progression. Nonetheless, the burden of the disease remains high, and while the improved survival of many patients has led to a shift in the ‘natural history’ to modified disease stages and phenotypes, it has not led to a cure [6, 53]. Indeed, it is clear that ERT is unable to address all aspects of this disease, particularly in terms of those etiologies associated with neuronal glycogen deposition [3, 47–51]. Crucially, substantial morbidity persists, and, particularly in infants, mortality rates remain high at 28–43% [53–56]. Beyond an initial improvement, most patients continue to decline, albeit at a slower rate than untreated patients, and remain below population norms [29]. Due to the time required to administer infusions, ERT by itself is considered burdensome to patients and caregivers [57]; whether this affects patient adherence and satisfaction would also provide more context to the understanding of the overall burden of Pompe disease.

Retrospective studies are generally inadequate for collection of patient-reported humanistic data, such as HRQoL, which are associated with significant recall bias and limited to the preceding few weeks. It is important to note that the young ages of patients with IOPD necessitates the use of caregiver-reported instruments to accurately reflect the range and severity of the burden across the spectrum of the disease; however, such ‘observer-reported outcomes’ are themselves associated with bias. In addition, as the delay from diagnosis to treatment shortens, it is becoming increasingly difficult to capture the experience of untreated patients; in order to do this; it is likely to be necessary to study patients in countries that do not have access to ERT. Future collaborations with existing prospective studies, such as the International Pompe Survey and the Pompe Registry, may possibly enable further investigation of the impact of ERT on the overall disease burden. However, to understand the humanistic burden in detail, a long-term observational study, focused specifically on the humanistic burden of Pompe disease, may be necessary.

### Alternative therapeutic approaches

There are alternative therapies under development for Pompe disease that, if successful, may help address the unmet medical need and the ongoing burden on the everyday lives of patients and their families. These include second generation ERTs that aim to improve targeting and extracellular uptake of the recombinant protein, either alone or in combination with pharmacological chaperones, to improve bioavailability and reduce immunogenicity. Oligonucleotide therapeutics in the form of mRNA-delivered GAA or exon-including antisense RNA (for c.32-13 T > G splice site mutations in late-onset patients) are in development [58–60]. Furthermore, gene therapies (in vivo adeno-associated virus [AAV] and Adenovirus, and ex vivo Lentivirus) are another route of treatment, seeking to continuously and endogenously produce GAA in target tissues and possibly minimize anti-GAA immunogenicity [61–64].

Thus, these new approaches may have the potential to substantially improve patients’ clinical and humanistic burden of disease, by resolving signs, symptoms and progression previously resistant to treatment and thereby improving patients’ HRQoL and ability to perform ADL. Given the increasing recognition of the importance of understanding and capturing the patient experience from patient and medical communities, as well as regulatory and reimbursement authorities, it will be key for new therapies to demonstrate not only their ability to treat the clinical signs and symptoms, but also to improve the day-to-day lives of patients and their families [65–68].

## Conclusions

This systematic review demonstrates that LOPD has a substantial humanistic burden that is clearly associated with clinical disease progression; patients with more severe disease have increased fatigue, more pain, poorer QoL, and less ability to perform ADL. Although ERT has been shown to provide some benefit, patients remain well below population norms. While there is a reasonable amount of research published for LOPD, gaps remain, including a complete absence of information on treatment satisfaction or adherence, and little information on the humanistic burden in the absence of ERT. For IOPD, the lack of any published articles on the humanistic burden represents a significant gap in the understanding of the most severely affected patients. As such, these findings point to an ongoing unmet need in the understanding of Pompe disease. As new therapies are developed, it will be critical to capture patients’ experiences and to further study the effect that the disease, as well as the treatment, has on both the clinical and humanistic aspects of Pompe disease [69]. Only in this way, will we be able to fully understand the effect that Pompe disease – and any potential treatments – have on the overall lives of patients and their families.

## Abbreviations

6-MWT: 6-min walking test
AAV: Adeno-associated virus
ADL: Activities of daily living
BPI: Brief pain inventory
CNS: Central nervous system
EDS: Excessive daytime sleepiness
ERT: Enzyme-replacement therapy
FSS: Fatigue severity scale
FVC: Forced vital capacity
GI: Gastrointestinal
GSDII: Glycogen storage disease type II
HADS: Hospital anxiety and depression scale
HRQoL: Health-related quality of life
ICIQ-B-LF: International Consultation on Incontinence Questionnaire Bowel-Long Form
ICIQ-UI-SF: International Consultation on Incontinence Questionnaire Urinary Incontinence-Short Form
IMT: Inspiratory muscle training
IOPD: Infant onset Pompe disease
IPA: International Pompe Association
LOPD: Late-onset Pompe disease
LOTS: Late Onset Treatment Study
LSD: Lysosomal storage disease
MC: Medical center
MCS: Mental component score
MRI: Magnetic resonance imaging
NHP: Nottingham Health Profile
PCS: Physical component score
PIS: Pain interference score
PSQI: Pittsburgh Sleep Quality Index
PSS: Pain severity score
RCT: Randomized controlled trial
RHS: Rotterdam Handicap Scale
RSQ: Respiratory symptoms questionnaire
SD: Standard deviation
SF-36: Short-form 36
sp./y: score points per year
UK: United Kingdom
US: United States
VAS: Visual analog scale
